# Immunoglubolin dynamics and cancer prevalence in Tasmanian devils (*Sarcophilus harrisii*)

**DOI:** 10.1038/srep25093

**Published:** 2016-04-29

**Authors:** Beata Ujvari, Rodrigo Hamede, Sarah Peck, David Pemberton, Menna Jones, Katherine Belov, Thomas Madsen

**Affiliations:** 1 Centre for Integrative Ecology, Deakin University, Waurn Ponds, Victoria, Australia; 2 Faculty of Veterinary Science, University of Sydney, Sydney, New South Wales, Australia; 3 School of Biological Sciences, University of Tasmania, Hobart, Tasmania, Australia; 4 Save the Tasmanian Devil Program, The Department of Primary Industries, Parks, Water and Environment, Hobart, Tasmania, Australia; 5 School of Biological Sciences, University of Wollongong, Wollongong, New South Wales, Australia; 6 School of Molecular Biosciences, University of Sydney, NSW 2006, Australia

## Abstract

Immunoglobulins such as IgG and IgM have been shown to induce anti-tumour cytotoxic activity. In the present study we therefore explore total serum IgG and IgM expression dynamics in 23 known-aged Tasmanian devils (*Sarcophilus harrisii*) of which 9 where affected by Devil Facial Tumour Disease (DFTD). DFTD is clonally transmissible cancer that has caused massive declines in devil numbers. Our analyses revealed that IgM and IgG expression levels as well as IgM/IgG ratios decreased with increasing devil age. Neither age, sex, IgM nor IgG expression levels affected devil DFTD status in our analyses. However, devils with increased IgM relative to IgG expression levels had significantly lower DFTD prevalence. Our results therefore suggest that IgM/IgG ratios may play an important role in determining devil susceptibility to DFTD. We consequently propose that our findings warrant further studies to elucidate the underpinning(s) of devil IgM/IgG ratios and DFTD status.

Since its first sighting in 1996, the Tasmanian Devil Facial Tumour Disease (DFTD) has caused massive (>85%) population declines of Tasmanian devils (*Sarcophilus harrisii*), hence questioning the long-term survival of this iconic species^1^. This highly contagious and clonally transmissible cancer is spread among individual devils via biting during social interactions^2^. DFTD cells are able to avoid host immune recognition by down-regulating MHC expression^3^. Metabolic failure, tumour related cachexia and metastases result in devil death within 6 to 9 months of the emergence of the first lesions^2^.

Presently, no therapy to reduce the devastating effects of DFTD has been developed. Numerous studies have, however, demonstrated that IgM antibodies provide extended tumour immunosurveillance as well as anti-tumour cytotoxic activity in other organisms^4^,^5^,^6^,^7^,^8^,^9^, and IgM antibody therapy has been shown to reduce neuroblastoma and melanoma in humans^10^,^11^. In the present study we therefore investigate the effects of total serum IgG and IgM antibody expression dynamics on DFTD prevalence in the world’s largest surviving marsupial carnivore; the Tasmanian devil.

## Results

**Figure 1:**
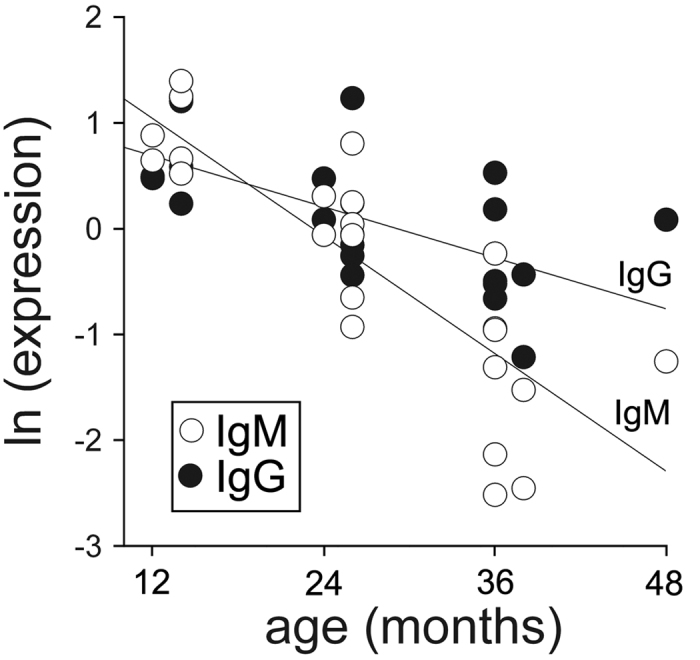
Relationship between IgM and IgG expression level and devil age.

**Figure 2:**
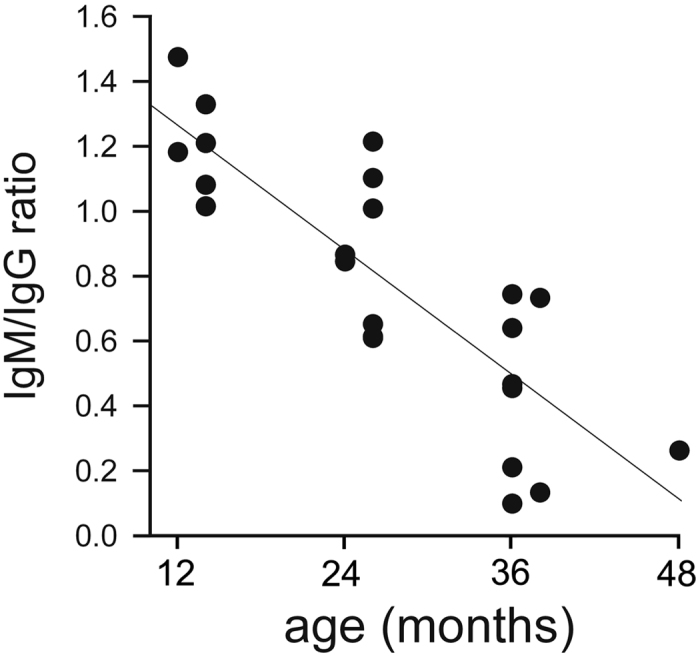
Relationship between IgM/IgG expression ratio and devil age.

Total serum IgM and IgG expression levels decreased with increasing devil age, and a single factor heterogeneity of slopes test with antibody as factor, age as covariate and antibody expression as dependent variable revealed a steeper age-specific decline in IgM compared to IgG expression levels (F_3,42_ = 24.24, p < 0.0001; age: F_1_ = 57.46, p < 0.0001; antibody: F_1_ = 6.29, p = 0.016; slope: age*antibody: F_1_ = 9.0, p = 0.0046, Fig. 1). Moreover, using a simple linear regression our analyses also revealed that IgM/IgG expression ratios decreased with increasing devil age (r^2^ = 0.70, p < 0.0001, N = 23; Fig. 2).

**Figure 3:**
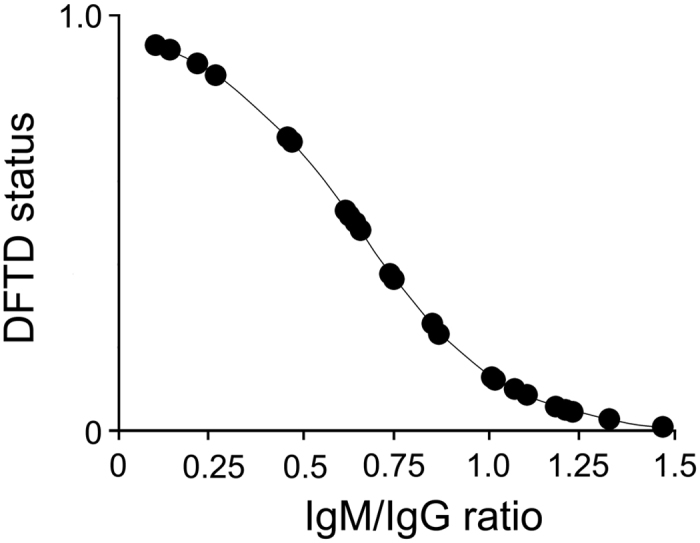
Logistic relationship between Tasmanian devil DFTD status (1 = infected; 0 = uninfected) and I gM/IgG expression ratio.

Using logistic regression we subsequently investigated whether the independent effects of devil age, sex, IgG and IgM expression as well as IgM/IgG ratio, and any of the possible two-way interactions between the five factors affected devil DFTD status. Following Quinn and Keough^12^ backward elimination was set at P > 0.2 which revealed that only devil IgM/IgG expression ratio had a significant effect on Devil DFTD status (Wald test, final model IgM/IgG ratio: χ2 = 5.90, p = 0.015, df = 1). Thus, devils with increased IgM relative to IgG expression levels had significantly lower DFTD prevalence (Fig. 3).

## Discussion

IgM antibody titers have been shown to increase with advancing age in numerous vertebrates and IgM dynamics has therefore been suggested to play a crucial role in maintaining immunocompetence during the ageing process^13^. The decline of IgM expression levels with increasing age suggests that devils are subjected to a significant age-related decline in immune function.

Although an experimental study showed that some devils are able to mount a specific IgG immune response to DFTD^14^ we did not observe any significant effects of IgG expression levels on DFTD status. As mentioned above, IgM antibodies have been shown to induce anti-tumour cytotoxic activity but in spite of this we did not detect any association between devil IgM expression levels and devil DFTD status.

DFTD prevalence has been shown to increase with increasing devil age^1^. However, the results from the present study demonstrate that only IgM/IgG expression levels, and not age, had a significant impact on devil DFTD status. In humans IgM/IgG antibody ratio has been shown to play a crucial role in resistance to diseases such as cerebral small vessel disease^15^. Moreover, in mice challenged by melanoma cells, increased IgM relative to IgG titers resulted in regressed tumour growth whereas decreased IgM relative to IgG titers caused aggressive tumour progression^4^. Although we can not rule out that DFTD results in a reduction in IgM relative to IgG titers, the results from the present study mirror those obtained in mice i.e. increased IgM relative to IgG expression levels significantly reduced devil DFTD prevalence, highlighting the importance of IgM/IgG ratios in cancer aetiology. We consequently propose that our findings warrant further studies to elucidate the mechanisms of total IgM/IgG ratios and devil DFTD prevalence.

Although active immunotherapy has not yet become the “magic bullet” in cancer treatment^16^, the results from the present study suggest the importance of IgM/IgG ratios in devil carcinogenesis. The development of anti-tumour vaccines that enhance the production of IgM relative to IgG antibodies and/or direct treatment with IgM antibodies such as PAT-SM6^11^ may therefore become an important component in combating the devastating effects of DFTD on devil population demography.

## Materials and Methods

### Animals, age and DFTD scoring

Twenty three devils (10 females and 13 males) were blood sampled (500 μl/sample) in March and May 2012 at “West Pencil Pine”, 15 km to the west of Cradle Mountain National Park in north-west Tasmania. Devil age was determined by using 20^th^ of March as the mean birth date and represented 8 age groups ranging from 12 to 48 months (for further details on aging see Hamede *et al*.^17^). Disease status was assessed by visual inspection of tumours and/or by histopathological examination of biopsies collected from tumours^17^. Of the 23 devils, 14 showed no clinical signs of being infected whereas 9 were confirmed to be infected by DFTD. The methods were carried in accordance with the approved guidelines of the University of Tasmania’s Animal Ethics Committee (A0010296), and all experimental protocols were approved by the University of Tasmania’s Animal Ethics Committee (A0010296).

### RNA extraction and quantifying IgM and IgG expression by quantitative RT-PCR (qRT-PCR)

RNA was extracted from blood samples using the RNeasy Protect Animal Blood Kit (Qiagen, Germantown, MD). Genomic DNA was removed from the RNA samples by the DNAse I AMPD1 kit (Sigma, St. Louis, MO) and cDNA was synthesized with the QuantiTect Reverse Transcription Kit (Qiagen, Germantown, MD).

Immunoglobulin heavy chain constant region segments were identified and sequenced as part of a previous study^18^ from the whole genome sequence of the Tasmanian devil available in the Ensembl database (DEVIL7.0, GCA_000189315.1)^19^. Only one transcript variant of IgM and IgG heavy chain constant regions have been identified the Ensembl database (DEVIL7.0, GCA_000189315.1)^19^. Gene specific primers were designed across exons of IgG (exon3 – exon4) and IgM (exon1 – exon2) heavy chain constant regions to avoid amplification of genomic DNA. The amplified cDNA products were cloned (using Topo-Ta Cloning (ThermoFisher Scientific, MA, USA) and sequenced using Sanger Sequencing to verify that functional IgM, IgG and RPS29 transcripts were amplified. The sequences have been deposited to GenBank under accession numbers: Saha-IgG: KU664590, Saha-RPS29: KU664591 and Saha-IgM: KU664592. Additionally melt curve analyses were conducted to confirm the lack of genomic DNA and primer dimers in the reaction (Supplementary Material).

cDNA was amplified without using primers from variable region sequences, thus allowing for a representative sampling of immunoglobulin genes expressed by circulating B cells. Our results therefore provide a “snapshot” of the levels of circulating IgG and IgM antibodies at the time of sampling.

Specific IgG and IgM primers were designed using the Primer3Plus website (http://www.bioinformatics.nl/cgi-bin/primer3plus/primer3plus.cgi); IgG primers: qIgG-F: 5′-CAG GTG ATC AGC ACT CTC TCT G-3′, qIgG-R: 5′-GGA TGT GGG GC AAG ACA TA-3′, IgM primers: qIgHM-F: 5′-TTT GAT ATC TGG GGC AAA GG-3′, qIgHM-R: 5′-ACA GCA AAG GAG GCA TCT TC-3′. To control for the potential of cell-type specific gene expression of reference genes three housekeeping genes were tested, glyceraldehyde 3-phosphate dehydrogenase (GAPDH), ornithine decarboxylase antizyme (OAZ) and 40S ribosomal protein S29 (RPS29) by using the software package BestKeeper^20^ which generates a Bestkeeper index by calculating and combining the geometric mean of the cycle threshold values (Ct values) for each sample across all housekeeping genes. Pearson correlation coefficients were subsequently used to compare each individual gene in a pair-wise fashion to the BestKeeper index, followed by ranking the candidate genes in order of their stability. The highest ranked gene is the most stable, and most suitable to be used as normalizer gene. To determine the most stable housekeeping gene, 7 randomly chosen samples were run with each housekeeping genes, GAPDH, OAZ and RPS29, and the obtained Ct values were analyzed by using the Bestkeeper software. RPS29 had the highest correlation coefficient (r): 0.981 (GAPDH r: 0.951, OAZ r: 0.694), therefore we ranked that gene the highest and used it as the reference gene in the subsequent analyses.

Similar to the IgG and IgM primers, gene specific primers were designed across exons of RPS29 (exon1 – exon2, only one transcript identified): RPS29-F: 5′-ATG GGT CAT CAG CAG CTC TAC-3′, RPS29-R: 5′-AGG CCG TAT TTG CGG ATT AG-3′). Quantitative RT-PCR was conducted on the RotorGene6000 (Qiagen, Germantown, MD). Standard curves (r^2^ > 0.99) contained five dilutions from the dilution series with a linear dynamic range of at least 3 orders of magnitude. PCR efficiencies ranged between 0.94 and 1.07 (IgG: 0.94, IgM: 1.04 and RPS29: 1.00). All samples were run in quadruplicate, and all Cq values for unknowns fell within the linear quantifiable range of the appropriate standard curves. Controls containing no reverse transcriptase enzymes, and controls containing no cDNA samples were run alongside the reactions to confirm that no genomic DNA was amplified, and the lack of primer dimers in the reactions, respectively (Supplementary Material). The program REST^21^ was used to calculate the normalized fold change of target compared with the reference gene.

### Statistical analyses

IgG and IgM expressions levels were ln-transformed to normalize distributions and to equalize variance across age groups. Analyses were carried out using JMP version 5.1.

## Additional Information

**How to cite this article**: Ujvari, B. *et al*. Immunoglubolin dynamics and cancer prevalence in Tasmanian devils (*Sarcophilus harrisii*). *Sci. Rep*. **6**, 25093; doi: 10.1038/srep25093 (2016).

## Supplementary Material

Supplementary Information
