# Supplementary material for: Immunoglubolin dynamics and cancer prevalence in Tasmanian devils (Sarcophilus harrisii)
Source: Sci Rep. 2016 Apr 29;6:25093. doi: 10.1038/srep25093 (PMC4850387; doi:10.1038/srep25093)
Supplement: Supplementary Information [file srep25093-s1.doc]

# Supplementary material

**Immunoglubolin dynamics reduce cancer prevalence in Tasmanian devils (*Sarcophilus harrisii*)**

Beata Ujvari^1,2^, Rodrigo Hamede^4^, Sarah Peck^2,5^, David Pemberton^5^, Menna Jones^4^, Katherine Belov^2^, Thomas Madsen^1,3,6*^

^1^Centre for Integrative Ecology, Deakin University, Waurn Ponds, Victoria, Australia

^2^Faculty of Veterinary Science, University of Sydney, Sydney, New South Wales, Australia

^3^School of Biological Sciences, University of Wollongong, Wollongong, New South Wales, Australia

^4^School of Biological Sciences, University of Tasmania, Hobart, Tasmania, Australia

^5^Save the Tasmanian Devil Program, The Department of Primary Industries, Parks, Water and Environment, Hobart, Tasmania, Australia

^6^School of Molecular Biosciences, University of Sydney, NSW 2006, Australia

# Quantitation report and melt curve analyses of the IgG standard curve RT-PCR reaction.

| Run On Software Version | Rotor-Gene 2.0.2.4 |
| --- | --- |
| Run Signature | The Run Signature is valid. |
| Gain Green | 9.67 |
| Threshold | 0.2992 |
| Left Threshold | 1.000 |
| Standard Curve Imported | No |
| Standard Curve (1) | conc= 10^(-0.287*CT + 8.239) |
| Standard Curve (2) | CT = -3.483*log(conc) + 28.694 |
| Reaction efficiency (*) | 0.93701 (* = 10^(-1/m) - 1) |
| M | -3.48272 |
| B | 28.69427 |
| R Value | 0.99931 |
| R^2 Value | 0.99861 |
| Start normalising from cycle | 1 |
| Noise Slope Correction | Yes |
| No Template Control Threshold | 0% |
| Reaction Efficiency Threshold | Disabled |
| Normalisation Method | Dynamic Tube Normalisation |
| Digital Filter | Light |
| Sample Page | IgG |

#### 1. Quantitation data for Cycling A.Green - IgG


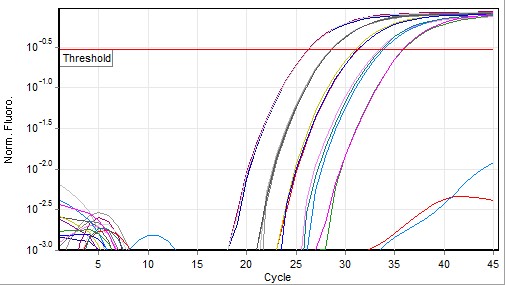


#### 2. Standard Curve - IgG


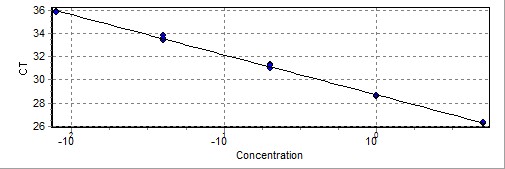


3. Standard curve data

| No. | | Colour | | Name | | Type | | Ct | Given Conc (ng/ul) | | Calc Conc  (ng/ul) | | | % Var |
| --- | --- | --- | --- | --- | --- | --- | --- | --- | --- | --- | --- | --- | --- | --- |
| 1 | 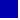 | | CTRL_5ng | | Standard | | 26.35 | | | 5 | | 4.72 | 5.7% | |
| 2 | 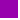 | | CTRL_5ng | | Standard | | 26.25 | | | 5 | | 5.03 | 0.6% | |
| 3 | 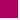 | | CTRL_5ng | | Standard | | 26.22 | | | 5 | | 5.13 | 2.5% | |
| 4 | 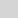 | | CTRL_5ng | | Standard | | 26.29 | | | 5 | | 4.89 | 2.2% | |
| 5 | 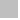 | | CTRL_1ng | | Standard | | 28.54 | | | 1 | | 1.11 | 11.0% | |
| 6 | 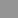 | | CTRL_1ng | | Standard | | 28.60 | | | 1 | | 1.06 | 6.5% | |
| 7 | 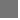 | | CTRL_1ng | | Standard | | 28.68 | | | 1 | | 1.01 | 1.1% | |
| 8 | 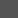 | | CTRL_1ng | | Standard | | 28.67 | | | 1 | | 1.02 | 1.7% | |
| 9 | 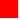 | | CTRL_0.2ng | | Standard | | 31.32 | | | 0.2 | | 0.18 | 12.0% | |
| 10 | 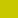 | | CTRL_0.2ng | | Standard | | 31.01 | | | 0.2 | | 0.22 | 8.4% | |
| 11 | 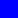 | | CTRL_0.2ng | | Standard | | 31.27 | | | 0.2 | | 0.18 | 8.7% | |
| 12 | 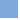 | | CTRL_0.2ng | | Standard | | 31.20 | | | 0.2 | | 0.19 | 8.5% | |
| 13 | 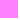 | | CTRL_0.04ng | | Standard | | 33.41 | | | 0.04 | | 4.42E-02 | 10.5% | |
| 14 | 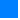 | | CTRL_0.04ng | | Standard | | 33.86 | | | 0.04 | | 3.28E-02 | 17.9% | |
| 15 | 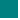 | | CTRL_0.04ng | | Standard | | 33.58 | | | 0.04 | | 3.95E-02 | 1.3% | |
| 16 | 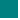 | | CTRL_0.04ng | | Standard | | 33.60 | | | 0.04 | | 3.99E-02 | 1.5% | |
| 17 | 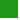 | | CTRL_0.008ng | | Standard | | 35.95 | | | 0.008 | | 8.28-03 | 3.5% | |
| 18 | 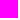 | | CTRL_0.008ng | | Standard | | 35.89 | | | 0.008 | | 8.57E-03 | 7.1% | |
| 19 | 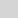 | | CTRL_0.008ng | | Standard | | 35.85 | | | 0.008 | | 8.37E-03 | 4.5% | |
| 20 | 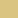 | | CTRL_0.008ng | | Standard | | 35.94 | | | 0.008 | | 8.18E-03 | 6.1% | |
| 21 | 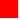 | | NTC | | No Template Control | |  | | |  | |  |  | |
| 22 | 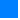 | | NTC | | No Template Control | |  | | |  | |  |  | |
| 21 | 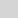 | | NTC | | No Template Control | |  | | |  | |  |  | |
| 22 | 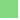 | | NTC | | No Template Control | |  | | |  | |  |  | |
| 23 | 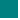 | | RT- | | Reverse Transcriptase (RT) Negative Control | |  | | |  | |  |  | |
| 24 | 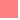 | | RT- | | RT Negative Control | |  | | |  | |  |  | |
| 25 | 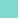 | | RT- | | RT Negative Control | |  | | |  | |  |  | |
| 26 | 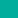 | | RT- | | RT Negative Control | |  | | |  | |  |  | |

#### 4. Melt curve for Melt A.Green –IgG


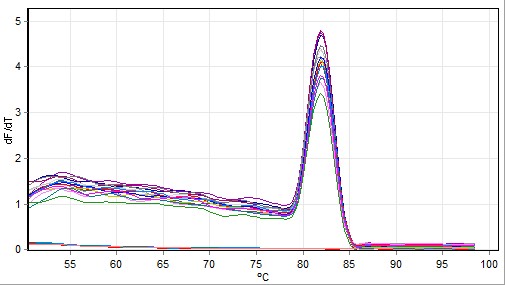


#### Quantitation data for Cycling A.Green - IgM


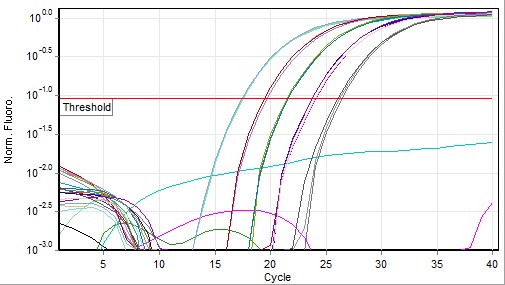


#### Standard Curve - IgM


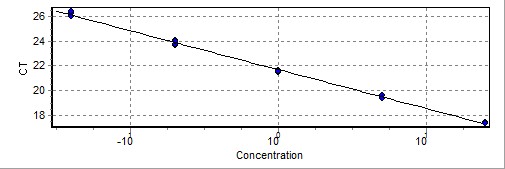


#### Melt curve for Melt A.Green - IgM


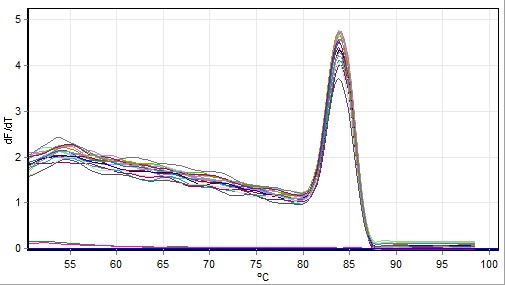


| No. | Colour | Name | Type | Ct | Given Conc (ng/ul) | Calc Conc (ng/ul) | % Var | |
| --- | --- | --- | --- | --- | --- | --- | --- | --- |
| 1 | 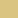 | CTRL_25ng | Standard | 17.46 | 25 | 22.84 | | 8.6% |
| 2 | 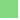 | CTRL_25ng | Standard | 17.43 | 25 | 23.24 | | 7.0% |
| 3 | 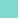 | CTRL_25ng | Standard | 17.48 | 25 | 22.39 | | 10.4% |
| 4 | 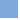 | CTRL_25ng | Standard | 17.38 | 25 | 24.17 | | 3.3% |
| 5 | 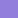 | CTRL_5ng | Standard | 19.66 | 5 | 4.49 | | 10.2% |
| 6 | 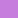 | CTRL_5ng | Standard | 19.42 | 5 | 5.37 | | 7.4% |
| 7 | 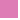 | CTRL_5ng | Standard | 19.41 | 5 | 5.41 | | 8.1% |
| 8 | 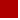 | CTRL_5ng | Standard | 19.42 | 5 | 5.34 | | 6.9% |
| 9 | 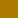 | CTRL_1ng | Standard | 21.60 | 1 | 1.07 | | 7.1% |
| 10 | 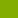 | CTRL_1ng | Standard | 21.46 | 1 | 1.18 | | 18.3% |
| 11 | 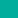 | CTRL_1ng | Standard | 21.57 | 1 | 1.09 | | 9.4% |
| 12 | 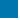 | CTRL_1ng | Standard | 21.59 | 1 | 1.08 | | 7.7% |
| 13 | 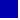 | CTRL_0.2ng | Standard | 23.65 | 0.2 | 0.23 | | 17.5% |
| 14 | 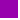 | CTRL_0.2ng | Standard | 23.70 | 0.2 | 0.23 | | 13.5% |
| 15 | 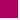 | CTRL_0.2ng | Standard | 24.02 | 0.2 | 0.18 | | 10.3% |
| 16 | 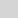 | CTRL_0.2ng | Standard | 23.97 | 0.2 | 0.19 | | 7.4% |
| 17 | 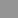 | CTRL_0.04ng | Standard | 26.18 | 0.04 | 3.62E-02 | | 9.4% |
| 18 | 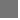 | CTRL_0.04ng | Standard | 26.37 | 0.04 | 3.16E-02 | | 21.2% |
| 19 | 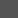 | CTRL_0.04ng | Standard | 26.01 | 0.04 | 4.13E-02 | | 3.3% |
| 20 | 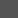 | CTRL_0.04ng | Standard | 26.10 | 0.04 | 3.77E-02 | | 5.4% |
| 21 | 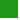 | NTC | No Template Control |  |  |  | |  |
| 22 | 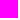 | NTC | No Template Control |  |  |  | |  |
| 23 | 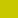 | NTC | No Template Control |  |  |  | |  |
| 24 | 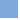 | NTC | No Template Control |  |  |  | |  |
| 25 | 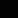 | RT- | Reverse Transcriptase (RT) Negative Control |  |  |  | |  |
| 26 | 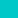 | RT- | RT Negative Control |  |  |  | |  |
| 27 | 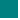 | RT- | RT Negative Control |  |  |  | |  |
| 28 | 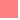 | RT- | RT Negative Control |  |  |  | |  |
